# Religious practices of Muslim women in the UK during maternity: evidence-based professional practice recommendations

**DOI:** 10.1186/s12884-022-04664-5

**Published:** 2022-04-19

**Authors:** Shaima M. Hassan

**Affiliations:** 1 Department of Primary Care and Mental Health, Institute of Population Health, University of Liverpool, Liverpool, L69 3GL UK; 2 NIHR Applied Research Collaboration NWC, Liverpool, England

**Keywords:** Muslim women, Religious practice, Maternity, Healthcare professionals

## Abstract

**Background:**

Muslim women commonly observe certain religious practices during their maternity journey and research in this area suggests that more could be done from a service provision perspective to support Muslim women in the UK through this significant life event.

**Aim:**

This study identifies Muslim women’s religious practices during maternity, needs and challenges of religious practice while engaging with maternity services, and support needs from healthcare professionals.

**Method:**

Qualitative mixed method study; that includes eight longitudinal interviews with first-time pregnant Muslim women, five focus groups with 23 Muslim mothers experiencing childbirth in last three years in UK, and 12 one-to-one interviews with Health care Professionals (HCPs) with previous experience working with Minority Ethnic groups. Participants recruited from local Muslim community groups and Maternity Care Provider, North West Coast, England. Data analysed using thematic analysis.

**Result:**

Qualitative findings indicate common religious practices that Muslim women exercise at different stages of their maternity journey. These practices can be divided into two categories of common religious practices for Muslim women that 1) require only healthcare professionals’ awareness of these practices and 2) require awareness and active involvement of healthcare professionals. Findings highlight key recommendations for healthcare professionals when addressing Muslim women’s religious needs in the UK.

**Discussion/Conclusion:**

This study provides evidence-based recommendations for professional practice to assist healthcare professionals in developing understanding and addressing Muslim women’s religious practice needs in the UK. Further research is required to explore the impact of these recommendations for professional practice.

## Background

Over recent years the UK has focused on enhancing the quality of maternity care and has encouraged a personalised and woman-centred approach that acknowledges that every woman is different in terms of her needs (cultural, religious and social) and choices [1]. Moving forward with the practical application of this approach is important given the growing diversity of the UK population.

The Muslim population makes up the second largest religious group in the UK and this population continues to increase [2]. Even though Muslims living in the UK are not a single homogenous group (being instead culturally, ethnically and linguistically diverse communities) [3], an overwhelming majority of Muslims in the UK will respect the rites of passage recommended by Islamic teaching [4]. Research has highlighted that Islamic beliefs and practices were at the core of Muslim women’s maternity experiences [5]. Whilst these practices provided a resource for Muslim women when faced with struggles during childbirth, many Muslim women lacked confidence to express their religious needs when engaging with maternity healthcare services [5]. A recent review of Muslim women’s experiences of maternity services in the UK highlighted that healthcare professionals appeared insensitive to Muslim women’s needs due to a lack of understanding of the religious values and practices, which impacted Muslim women’s confidence in discussing their specific needs [6].

This finding demonstrates the importance of understanding the religious practices during maternity of Muslim women and their impact within maternity services. Rassool suggests that healthcare professionals who have an understanding of Muslims’ worldview and religious/cultural practices are better placed to provide woman-centred care [7]. Swihart and Martin highlight the importance of empowering healthcare professionals with the knowledge and skills to respond to the religious/cultural needs of patients and their families [8]. Hasnain et al., also reported that whether in the United States or Western countries, improving care for Muslim women would require a care model that included training health care professionals and made necessary adjustments in the healthcare system to accommodate for women’s needs [9].

This paper describes Muslim women’s religious practices during maternity, through both the perspective and experiences of Muslim women and healthcare professionals, to provide evidence-based recommendations to help assist healthcare professionals when providing woman-centred care for Muslim women. The aim is enhancing awareness and understanding of Muslim women’s needs amongst healthcare professionals, encourage better interaction and build Muslim women’s confidence in expressing their needs.

## Methods

This paper presents findings from a three-phase qualitative study that explored Muslim women’s experiences of motherhood while engaging with NHS maternity services [5, 10]. A qualitative research design offered flexibility to Muslim women and HCPs in discussing their perceptions and experiences of Muslim birth practices. Having both perspectives created a deeper insight into how religious practices are acknowledged in relation to maternity services.

### Participant recruitment

There were three phases of participant recruitment to the study, the details of each phase is highlighted in Table 1.

**Table 1:** Study research design and recruitment

Research phase	Approach and sample	Setting and population
Phase One	Eight longitudinal semi-structured interviews—each included three interviews at different time points of maternity care continuum: (i) prenatal (29 to 40 weeks of pregnancy), (ii) immediate postnatal (within the first two months after birth) and (iii) later postnatal (four months after birth).	First time pregnant Muslim women recruited from local Muslim community groups e.g. mosque and Muslim community groups.
Phase Two	A total of 23 Muslim mothers were recruited to take part in one of five semi-structured focus groups.	Muslim mothers having experienced childbirth in the last three years in the UK, recruited from Muslim women groups in the local community e.g. ‘mother and toddler’ and breastfeeding groups.
Phase Three	One-to-one interviews with 12 HCPs: Midwives in a variety of specialist roles (7), Gynaecology Nurses (2), Breastfeeding Support Workers (2) and a Sonographer (1).	HCPs recruited through a large maternity provider located with the North West of England.

### Data collection

One-to-one and focus groups interviews were conducted between 2013 and 2016 in locations suitable for each participant, this included women’s own homes, local community centres, women’s group settings, maternity care Trust and coffee shop. Interviews and focus groups were approximately 60–90 min long. A semi-structured interview guide was used that included open questions that focused on Muslim women, religious practice and how this practice influenced maternity experiences and how these experiences can be acknowledged and addressed. Recruitment and interviewing continued until data saturation was achieved; that is, when no new information emerged from new data collected. Participants provided written informed consent prior to interview.

### Data analysis

Audio-recordings were transcribed verbatim by (SH) and reviewed by the study’s supervisors (CL), (KB) and (JR) for consistency and credibility. Data from each phase of the study was analysed using a structured thematic analysis approach [11] to identify, analyse and report key themes within the data. All main themes from across the three phases were combined to identify key overarching recommendations. The themes identified in this three-phase study included; Muslim women’s spiritual perspective; Muslim women’s expression of religious requirements; HCP’s perceptions about Muslim women; HCP’s understanding and awareness of religious practices; HCP’s approaches in addressing and supporting Muslim women’s religious needs; Importance of training in providing culturally and religiously appropriate woman-centred care which are reported elsewhere [5, 10]. This paper’s analysis focused on themes identified highlighting Muslim women’s religious practice and presented these findings in detail building on recommendations that provide detailed insight to Muslim women’s religious practices in the UK. Final identified recommendations were reviewed by the supervision team, two healthcare professional and a public adviser (Muslim woman).

### Ethical consideration

The ethic approval was obtained from the NHS Research Ethics Committee (through the Integrated Research Application System (IRAS) [REC reference: 13/WS/0087; IRAS project ID: 117529]) prior to commencing data collection. Written consent was obtained from all participants in the study.

## Results

Forty-three participants discussed Muslim women’s birth practices and their implications when engaging in maternity care services. When participants were asked about their religious practices during their motherhood journey they discussed different aspects such as the religious practices recommended by religion, the practices that they planned to implement, and the practice that they were unable to implement. All religious practices reported by all participants in this study and in which period of maternity they were practiced are highlighted in Table 2. Note, all participants are given a code or a pseudonym name.

**Table 2:** Religious practices mentioned by participants

Religious Practice	During Pregnancy	During Labour	During Post-labour
*Recitation of Quran and Supplications*	√	√	√
*Maintaining modesty*	√	√	√
*Absences of male health professionals*	√	√	√
*Fasting the month of Ramadan*	√		√
*Dates as pain relief*		√	
*Silent birth*^*a*^		√	
*Burying of placenta*			√
*Adhan and Iqamah*^*b*^			√
*Tahneek*^*c*^			√
*Animal-based product in pharmaceuticals*^*d*^	√	√	√
*Breastfeeding*			√
*Male Circumcision*			√
*Shaving the hair of a new born*			√
*Aqiqah*^*e*^			√
*Community visiting mother after childbirth*			√

Details of each religious practice is highlighted in Tables 3 and 4

^a^ Silent birth – Some Muslim women prefer the first word that their child hears at birth to be Allah’s name or the word of Allah. So, everyone attending the birth should refrain from spoken words as much as possible

^b^ Adhan and Iqamah—To whisper the Islamic call for prayer to the new-born soon after birth

^c^ Tahneek—A small piece of softened date (sometimes honey) being gently rubbed into the child’s mouth (on the upper palate)

^d^ Main prohibited foodstuffs and liquids include Pork (and its by-products); animal fats and meat that has not been slaughtered according to Islamic teachings and alcohol

^e^ Aqiqah—Seven days after birth, a sheep is offered in sacrifice and the meat is distributed among family members and the poor within the community

### Recitation of Quran and supplications

The recitation of the Quran is the first and most constant religious practice carried out by participants throughout the stages of childbirth. Through the recitation mothers reflect on Allah’s words as a form of worship. All participants had plans for the Quran recitation to be played using an audio device during the early stages of their labour. Some requested a CD player from their midwives to play the Quran recitation in the room they are staying in during Labour and some used their own devises such as using a mobile phone. Some had Muslim birth partners or sought Muslim healthcare professionals to assure that the religious practices were fulfilled.

*‘I wanted the recitation to be played and someone reciting certain supplications next to me. That is why I really wanted Muslim sisters to be there and not my non-Muslim family, because if I had gone in with my non-Muslim family and friends, I would have been too weak and I would have just been overpowered and none of it would have happened.’* (**Gp3; P3**).

‘*I remember a lady on antenatal clinic who used to play the Quran every evening just to calm herself down. Either play it or read it*’. (**HP-6**).

Through supplication mothers would ask Allah for ease at the time of their struggles and for the protection of their child. Some women mentioned certain attributes of Allah that they will call out during supplications that relate to their need, especially during labour, such as Al- Latif (The Gentle), Al-Karim (The Generous) and Al-Wadud (The Loving One).

‘*I remember my sister making me call it out and the midwives were watching me. I had to do it and my midwife was there and they said it was amazing*.’ (**Gp4; P2**).

### Maintaining modesty

In this study modesty was discussed by all participants; for the majority of participants the main concern was to maintain their modesty during labour and during examinations. It was important for them not to be too exposed during labour; for some, this concern was causing them anxiety as to whether they would be able to maintain it at all times, and whether their midwives would acknowledge this concern.

‘*For me I think modesty definitely ties in with my religion, you are not just going to let it go because you are having a baby, so you have to hold on to your belief*.’ (**Noor**).

Some healthcare professionals were aware of this and used available resources such as extra sheets to make sure the women feel comfortable during labour or clinical examination.

‘*I saw a lady a while ago who wore the full Burku, a face veil, which you might think would be difficult but I took her to a private room as she had difficulty with breastfeeding. She was very happy for me to remove her veil to see how the baby fed.’* (**HP-10**).

Sahar explains how her midwife recognised this need and acted to deliver it.

‘*When they put me on the wheelchair to go and get my stitches, I never had a head scarf and they were going to take me out of the room. I said to my husband can you please pass me my head scarf and I think that was the first time my midwife actually realized I am a Muslim. Then she said, “get the sheet and cover her (my) legs”. It is then that she made more of an effort.*’ (**Sahar**).

### Absences of male healthcare professionals

All mothers preferred not be seen by male healthcare professionals during scan appointments, clinical examinations or attending during their labour. Participants explained that religion gives them an exception during the unavailability of a healthcare professional of the same gender to be attended to by the opposite gender. However, participants had different opinions on how they would approach this situation; for some if a female is not available then they would take the religious exception, others would request their appointment to be rescheduled with a female, and some did not feel confident enough to ask about whether a female healthcare professional was available to attend to their care. They felt that the healthcare professionals would not acknowledge this need, and they might be considered as a burden through what they believed—that their need is an extra demand on the services.

‘*I was seen by a man healthcare professional, I actually felt very uncomfortable but I felt I like I was in a situation where I could not even say to him “can I have a woman” … I feel like he may think “Oh here we go, a Muslim woman complaining” or “Oh she is making more work on us*’ (**Samah**).

However, during labour some participants were in a situation where male healthcare professionals had to be involved for medical reasons. Some felt that they would only accept to be seen by a male professional once they were in a critical situation and they had insisted on being attended to by a female professional.

‘*The midwife told me there is only so much she can do but she needs to get the doctor in, then I said ok, can I not have a man. They went back and tried; there was a female doctor that just about come out of her shift then she just stayed and helped me through labour*.’ (**Gp4; P3**).

Some healthcare professional participants reported that they would try and explore if a Muslim woman was happy to be seen to by a male professional and try to give her the opportunity to express her preference.

‘*I say to them in a subtle way, we have got a male and a female doctor on today, so is that okay? And I give them that opportunity then to say to me, well, actually can I see the female? And I will say, yeah, that is fine, no problem. And I put a note on, female only. If I have two male doctors on, which does not happen too often, but if it does happen, I will say to them, both of our doctors today are male, how do you feel about that? And they are like, no, no, I'm not going to see a male. Or they might say, that is fine*.’ (**HP-8**).

### Fasting the month of Ramadhan

There was a difference in opinion amongst participants in whether they would observe the fast while pregnant or breastfeeding, even though their religion exempts them from observing the fast during this period. Most participants attempted to fast, if they felt that they were physically unable to tolerate the fast, they would then consider the exception. More than half of the participants said that Ramadhan is a spiritual month that all the community engages in, therefore it is difficult for one to make a choice to not engage in this community worship. The majority of health care professionals did not agree to fasting during pregnancy and some acknowledged and respected choice in fasting.

‘*I remember when I was pregnant it was Ramadhan and I was doing one day on and one day off of fasting… The midwife said to me “you know yourself and listen to your body” and “you can do what you want to do” and she respected that. It made me feel good that she respected*.’ (**Gp2; P4**).

Meanwhile, the majority of participants said that they would not mention the fast to their midwives. They explained that midwives would often advise against fasting during pregnancy. Healthcare professionals in this study reported that they would advise the women not to fast and reflect on the religious prospective, however, they felt that some women wouldn’t consider their advice.

*‘All you can say is, we prefer you not to fast in pregnancy, you are immune from fasting while you are pregnant or breastfeeding, so please do not do it. But I know that most women will still fast and some women miss the appointments because they are fasting’.* (**HP-4**).

‘*If only they have leaflets on fasting while pregnant or breastfeeding because it is not easy just to tell someone to not fast. So it is nice to have something that is fixable and just informs the mother on what she can do and eat to make sure that she is still healthy while fasting, like what food and drinks to have when breaking the fast*.’ (**Fatimah**).

### Eating dates during initial stages of labour

Eating dates during the early stages of labour is common amongst the majority of participants. They highlighted the eating of dates during the early stages of labour is religiously recommended, as it is considered as a form of pain relief and an energy source. The majority of participants started to eat dates while still at home as soon as labour pains came about, they also continued to have the dates during the early stages of labour in hospital. Mothers explained that this practice can be difficult to maintain during their following pregnancy, labour can often be very spontaneous and fast giving no time to have dates.

‘*Obviously when you pack the hand bag that you bring with you to the hospital, we make sure we take dates with us. So, before you look at science, if you are a person of religion you are going to look at how religion teaches you and you learn that it tells you to have dates to ease labour*.’ (**Salee**).

### Silent birth

This practice was mentioned by some participants; they explained that they preferred to have silent in the labour room when their child is born or for everyone attending the birth refraining from speaking words as much as possible. This is because they believed that the first words that their child should hear are the words of Allah. Some participants reported that their midwives acknowledged this when they mentioned it on arriving at the hospital. However, like most mothers, other participants did not feel confident enough to mention this practice to their midwife; one participant tried to compromise by calling out the name of Allah in a slightly higher voice then the voices in the room in order for her baby to hear the word of Allah first.

‘*That was something that I made sure of and that the midwives do not speak, and the first word to be Allah. I think my husband vocalised that too and said to the midwives “can we please have silence” and I think they would have completely respected it and they were really good like that.*’(**Noor**).

### Burying the placenta

The burial of the placenta was mentioned by all the participants as an Islamic recommended practice; they explained that as it is an obligation to bury the dead human body and it is encouraged to bury any separate part of the human body if possible out of respect. All the participants wished to do this practice but the majority found it difficult to practise without having the facilities. They explained that a common practice is that the placenta is buried in the garden of one’s home but because they do not have a garden and were not sure of any other option they have decided not to engage in this practice. Only two participants managed to do this practice and as for mothers only a few managed to bury their placenta.

‘*I put down that I want the placenta because of religious beliefs that we do not incinerate any human parts and I think in the hospitals they incinerate the placentas*.’ (**Noor**).

### Adhan and Iqamah [call of prayer]

This practice was considered as one of the most important practices carried out by mostly the birth partners; religiously it is recommended to whisper the Adhan [call of prayer] into the baby’s right ear and the Iqamah into the left ear. This is considered as a significant religious ceremony that is highly recommended to take place first thing when the child is born. Although the majority of participants and their birth partners were confident to implement this ceremony with the presence of the midwives in the room, they were not confident enough to fully inform or explain to the midwives what they were doing or intended to do. The majority of participants said that midwives or the staff that were in the room were busy completing what they had to do and did not notice the practice. Generally, all participants said that they would have appreciated less talking in the room while this was practised. Two participants reported their midwives acknowledged this and remained quiet while their partner completed the practice; meanwhile similar to some mothers from the focus groups and interviews, they had to delay this practice until the midwives had left the room.

‘*I wanted her to hear the Adhan for me and my husband that is so important. The baby was passed to my husband. So I said “we need to read something for the baby, can you please be quiet while we do that” they just said “ok” and they just continued talking and whispering. It was clear that they did not understand what we were doing*.’ (**Sahar**).

‘*We could not do it, we hid away. When my husband took the baby and went to one side, the midwife was like “where is he?” “What has he done with the baby?” And she kept on asking and she was like “we really need to clean him now” and she was trying to take the baby.*’ (**Gp4; P1**).

### Tahneek

This practice was another reason why the participants prepared dates to bring into hospital for labour. It is recommended that soon after the birth before the baby’s first feed, for the mother or the birth partner to take a small piece of softened date and gently rub it into the baby’s mouth. Some participants practised this as recommended, they had dates with them in the hospital and others practised once they arrived home after the baby’s first feed. Meanwhile all participants avoided doing this practice while midwives or staff were still present in the room; they explained that staff may consider this as taboo and would discourage it. So, to avoid being discouraged, all participants delayed this practice until no healthcare professional was present.

*‘I did not want them to think that I am not a good mother and say “look she is putting solids in his mouth”. I did not want them to take it to that extreme. So that was quite a personal time for me, I just wanted them to leave, so I can do it while they were busy*.’ (**Gp2; P3**).

Some participants mentioned that they read an article referring to scientific research that highlights the possible benefits of given a new-born sugar gel by rubbing it in the inside cheek of premature babies to protect against brain damage. Samah, Fatimah, Noor and other mothers said they find it more beneficial when science backs up their religious practice, as it helps in removing the taboo of the practice.

‘*My husband did the Tahneek when the midwife left the room because she would not understand. There is research that has just come out about giving a new-born child sugar can help protect them from brain issues. For the health professionals it is nice to have evidence to back up our practice*.’ (**Samah**).

### Animal-based products in pharmaceuticals

There are a large number of animal products in pharmaceuticals that can possibly present Muslims with a serious dilemma; weighing their health against their religious principles. This concern was raised by a few participants in this study when the Vitamin-K injection given to the child at birth has some animal derivatives that are unlawful for Muslims. Some tried to find another alternative for the Vitamin-K that is free from any animal derivatives and some accepted the injection as it is for the benefit of the child health.

*‘I mentioned that I did not want the Vitamin-K injection which was a big decision for me; I found out that the Vitamin-K actually has pig’s ingredients, which is completely prohibited in religion.*’ (**Noor**).

Some mothers from the focus groups were also in a similar situation and suggested that the maternity services should have a product free from animal derivatives available in the hospital. The majority of participants were not aware that the Vitamin-K injection may not comply with their religious beliefs and presumed that they would have been informed of animal-based pharmaceuticals because they believed that healthcare professionals would be aware of their dietary needs.

*‘I think the health professionals are well educated and I trust that. If there was something that is forbidden in other people’s religious or dietary needs, I think they are smart enough to tell us and it would be silly if they do not*.’ (**Hanan**).

### Breastfeeding

All participants in this study committed to breastfeeding. Participants were aware of the breastfeeding health benefits, however their commitment to breastfeed is mainly inspired through their religious teachings. Participants showed great understanding of the religious teachings regarding breastfeeding, they highlighted that in the Islamic traditions breastfeeding is a highly rewarded act, encouraging mothers to breastfeed her child for a maximum period of two years. Three participants explained that they considered the uptake of breastfeeding even though breastfeeding was not a common norm witnessed amongst their families.

*‘In terms of breastfeeding, the Quran speaks about the blessing and rewards of this act and even how long you should breastfeed for. The breast milk is pure and she is born a Muslim, where I was not. I want to give her the best start as much as Islamic influence as possible. I was concerned that I probably would not succeed in breastfeeding but I persevered because of the Islamic element. I understood that there is a reward and blessing in this act and that mainly pushed me to do it*.’ (**Sahar**).

There are many challenges in breastfeeding. Most mothers mentioned that they sought support from midwives, breastfeeding support team and family and friends while trying to establish breastfeeding with all their children. All participants explained that support during the early stages of breastfeeding is key in helping them persevere.

*‘I know a lot of girls see breastfeeding as a natural thing but the reality of it is that it is hard and the struggle of it is hard. Unless you have someone around you guiding and supporting you, I can imagine a lot of girls just quit*.’ (**Gp1; P1**).

However, healthcare professional participants fund that the majority of Muslim women would already have their mind set on breastfeeding and they are normally very good in initiating and maintaining breastfeeding. For some they believed that Muslim women have support within the community to help them in breastfeeding.

“*They will always know somebody who's breastfed. They have got women, very supportive women in the community, they have all breastfed before, someone's going to help them do it*”. (**HP-4**).

Participants found breastfeeding challenging in the presence of others or in public. The majority of participants said that they would stay home most of the time to avoid breastfeeding in public. Even in front of other women, some participants did not feel confident enough to breastfeed because they felt a bit exposed. Using a breastfeeding apron/cover helped some gain the needed amount of privacy with their baby while breastfeeding.

‘*I would like to breastfeed my own child indoors and I would not feel comfortable to go and breastfeed somewhere else. Even at the hospital ward I had to make sure that the curtain was always closed*.’ (**Khadija**).

### Male circumcision

This practice was discussed by three women from the interviews and many mothers from the focus groups who have had male children. The participants explained that this practice was an important religious requirement that has no religious exceptions and one cannot be laidback about. It is recommended for the male child to be circumcised as early as seven days after birth; all participants aimed for their children to be circumcised early as it is recommended but most found it difficult. All participants explained that they lacked information regarding how and where circumcision can be done. They were keen on seeking a safe and reliable circumcision clinic. Before making a decision on how or where to do the circumcision, mothers tried to source information from NHS services, family and friends, and Muslim healthcare professionals within the local community.

*I went to the children’s hospital and they do not do it early because of the risk of putting the baby to sleep. With my first, I did not want to go to a private surgery just in case anything goes wrong because with the NHS it has its standards and they will follow it up if anything goes wrong.*’ (**Gp4; P4**).

The majority said that the NHS was the first place they sought, however, difficulty in doing this practice in the NHS meant that some participants had to find other alternatives. Some were in two minds whether to wait on the NHS or consider private circumcision clinics, which often made them feel anxious. Some sought private NHS accredited clinics that were trustworthy. Others followed the recommendation of other people who had used the private clinic for their children.

‘*There are sisters that did not know that there are private clinics and they would wait for the NHS until their children are so much older, they are going to be in so much pain. Where I took my child it was so professional private doctor was amazing*.’ (**Gp3: P3**).

However, there were mothers that chose to take a different route the second time they had to go through circumcision. Some mothers had their first child circumcised in the NHS but chose to take their second child to a private clinic. They explained that they realized that the earlier the child is circumcised the better and the quicker the healing process is. Other mothers had their first child circumcised in a private clinic and then decided to have their second child circumcised on the NHS. Some said that the circumcision of their first was not done appropriately, which then caused them to end up in the NHS, so they decided to not make the same mistake and just waited for the NHS for their second child.

‘*My first boy was circumcised on the NHS and that was awful. I wish I did it when he was younger, all my sisters did it when their boys were 40 days and I wish did not wait until my child was a year. For my second one I am certainly going private and doing it within the first 40 days*.’ (**Gp2; P1**).

All participants explained that they had no form of information given to them by the health services; they explained that they would have benefited and felt supported if they were provided with information on circumcision from the NHS and signposted to private clinics that are accredited by the NHS. Some healthcare professional participants said there is some awareness in the services about male circumcision, which they thought is important to help clear the confusion amongst the staff and also to help them direct Muslim women if they were to inquire about this matter.

*‘I actually went to an event recently where there was some information about circumcision, which there is only a handful of centres that are certified. So I took the information and I thought, you know, in future if I get asked or if I wanted to send this information that this is what we can advise Muslim women that would be helpful*’ (**HP-6**).

### Shaving the hair of a new born

This was a practice that participants briefly discussed; traditionally on the seventh day of child’s life the scalp hair that has grown in utero is removed, and an equivalent weight in silver is given to charity. Some mothers engaged in this practice; once they were home the husband or a family member would shave the hair or bring someone to do so and distribute silver money that is equivalent to the weight of hair to the needy.

### Aqiqah

This is a practice that was implemented by all participants. In the Islamic tradition, a sheep is offered in sacrifice for every newborn child as a sign of gratitude to Allah. This is recommended to take place on the seventh day after the birth of the child and the meat is distributed among family members and the needy. Some participants did the Aqiqah in a form of a celebration meal; the sacrificed sheep was cooked and served to family members and friends. As for others, the sacrificed sheep was divided into portions and given to family member and neighbours.

### Community visits the mother after childbirth

It is a common tradition amongst Muslims to visit a mother after her birth; participants explained that visits start straight after birth and continue for two to three weeks. The purpose of these visits is to celebrate the coming of a new child and health of the mother. Visitors will bring food and gifts, and will sit with the mother for a friendly chat. Some participants said that these visits can be overwhelming, they explained that the first two weeks of the child’s life is the time for them to bond with their new born and get used to the changes that were happening in their lives. Some managed to send a message asking the community visiting to not visit in the first week after their birth, this gave them a chance to settle back home with their child. Meanwhile the others felt that it is impolite to stop people from visiting, they explained that it was a blessing to have people visiting you but it was difficult to maintain the demands of their child and hosting guests at the same time. Some participants stayed at their mother’s home and others had family members staying with them for support during this time. Most mothers praised this practice, they explained that it helped everyone to check on each other.

‘*Traditionally we have visitors come see the baby but I was not very keen about them because you need time to get used to the changes that happen in your life with the baby coming in to it. But when the guests came you have to be very formal, presentable to people and talk to them*.’ (**Fatimah**).

### Religious practices and writing a birth plan

Finally, on reflection on religious practice and participants ability to express their needs in relation to these practice during their engagement with maternity services, every woman was given the opportunity to discuss and write anything specific to be acknowledged during labour. This can include her choice of pain relief, where she would like to give birth and any specific practice that she would like the midwife to be aware of. A birth plan sheet is provided in the handheld notes; many participants were not aware of this sheet. Even though participants expressed many practices that they were keen on implementing, the majority did not prepare a birth plan sheet. Some said that they do not think that midwives would have a chance to look through their birth plan at the point of labour and some said that they were not sure if the midwives would understand their religious needs.

‘*My disappointing birthing plan appointment with my midwife that lasted 10 min of a simple tick list and just assumed things without asking me; Gas and air tick, information leaflet tick, birth at home no. She confused me so much that I forgot to mention some of the Islamic practices that I wanted to do during labour*.’ (**Sahar**).

Mothers did not mention that they prepared a birth plan; none of the mothers seemed to be keen on writing out a birth plan. When they were asked if they had written a birth plan, many looked confused and were unaware of what a birth plan was.

‘*It did not give me the option of mentioning any religious practice that I wanted to practise. Maybe it was not that black and white, maybe it was down to me to write it on there, but I do not think they have the time to look at it any way’*. (**Gp4; P1**).

## Discussion

The study identifies specific religious practices that Muslim women may engage with during their maternity journey and highlights their specific practice needs when engaging with maternity services. These practices and needs resulted in the development of practical recommendations to assist HealthCare Professionals (HCPs) to provide appropriate care for Muslim women.

Our findings add weight to existing evidence of the importance of enhancing person/woman-centred care that meets the needs of a growing diverse population in the UK. The Midwifery 2020 programme vision highlights the importance in maximising the potential for midwives to develop capacity and capability in delivering research-based practice in a changing environment [12]. The Five Year Forward View also suggests considering different approaches in how our maternity services need to change to meet the needs of the population [13]. Emphasising woman-centred care that prioritises the women’s individual needs that are defined by the women themselves, promoting choice, control and equitable care [14]. This proposition reflects the findings and recommendations of this study to contribute to enhancing maternity care for Muslim women and creating understanding of their specific needs as defined by them.

The findings of the study have practical importance given the evidence that minority ethnic women are still not receiving high quality maternity care for many reasons, which include judgmental and stigmatizing attitudes by health professionals [15], and are at higher risk of maternal mortality [16, 17]. Lack of understanding for example, of specific cultural/religious needs that may clash with certain medical routines, creates difficulties for ethnic minority women when engaging with maternity services [5]. Therefore, creating awareness and supporting healthcare professionals to enhance their understanding of cultural diversity is crucial for achieving an effective maternity service [3, 5, 6, 10].

The religious practices identified are organised into two categories of common religious practices for Muslim women that 1) require only healthcare professionals’ awareness of these practices (Table 3) and 2) require awareness and active involvement of healthcare professionals (Table 4). Final identified recommendations were reviewed by the supervision team, two healthcare professionals, an academic researcher and two public advisers (Muslim women).

**Table 3:** Common religious practices for Muslim women that require only healthcare professionals being aware of these practices

Common religious practice	Practice need	Recommendations for healthcare professional
**Supplications (*Dua’a*)**		
Tendency for verbal supplications—calling on Allah’s (God) name and His attributes usually in the Arabic language—seeking Allah's support at time of struggle.	**Awareness –** some Muslim women/birth partners may not feel confident to recite supplication if they feel that HCP has a lack of understanding of this practice.	**Awareness—**show understanding and acknowledgement of this practice.
**Eating dates (fruit) during the initial stages of labour**		
Imitating the action of Maryam (Mary the mother of Jesus) during her labour, some Muslim women eat dates for energy during the initial stages of labour.	**Acknowledge**—acknowledgment of the practice.	**Awareness**—some Muslim women will bring dates with them to hospital.
***Tahneek***		
Commonly practiced soon after the child is born, and preferably before the child’s first feed. A small piece of softened date (sometimes honey) being gently rubbed into the child’s mouth (on the upper palate). Some Muslim women bring dates to hospital so they can carry out this practice. Others delay this practice until they are home.	**Awareness –** some women my fear that HCPs may not approve of this practice, therefore, may do this once the HCP is out of the room.	**Awareness**—that some women may not feel confident to practice this in the presence of HCPs. While exploring the birth-plan with the woman and her preferences during labour, show understanding and acknowledgement of this practice.
**Shaving the head hair of a new born child**		
The scalp hair that has grown in utero is removed, traditionally on the seventh day of life, and an equivalent weight in silver is given to charity *Note: not all Muslims will engage in this practice.*	**Awareness—**general awareness and acknowledgment of the practice.	**Awareness—**that some Muslim parents would engage in this practice **Acknowledgement** – be mindful that some mothers may not feel confident to answer genuine comments made by HCPs, such as ‘*why did/would you shave his/her beautiful hair*?’
***Aqiqah***		
Normally seven days after birth, a sheep is offered in sacrifice and the meat is distributed among family members and the poor within the community. Some distribute the sacrifice as cooked food in a family gathering whilst others pay for sacrifice to take place in a country other than the UK to be distributed to the poor.	**Awareness—**general awareness.	**Awareness**—general awareness.
**Congratulating and community visits mother after childbirth**		
It is common for mothers to receive visits from others within the community soon after birth. The purpose of these visits is to celebrate the coming of a new child and health of the mother. Visitors will bring food and gifts, and will sit with the mother for a friendly chat.	**Awareness –** These visits can start at hospital and continue at the women’s home. Mothers often receive gifts, cooked food, and get to hear the experiences and advice of other women regarding motherhood.	**Awareness—**Be aware that some Muslim mothers may have lots of visitors, some arriving at hospital shortly after birth. **Acknowledge the mother –** whilst this practice can be joyful for mothers, sometimes it can be overwhelming having lots of visitors at hospital or at home, especially for first-time mothers or mothers recovering from medical procedures. It is important to check on the mother and ensure that she is well.

**Table 4:** Common religious practices for Muslim women that require awareness and active involvement of healthcare professionals

Common religious practice	practice need	Recommendations for healthcare professional
**Prayer**		
Muslims have an obligation to complete five daily prayers, performed at different times of the day. Prayers include physical movement—standing to face the South East (toward Macca), bowing and prostration.	**A prayer space**—The woman/partner may need to perform the prayer while waiting for their appointment or during their stay in hospital. **A washing facility**—Muslims perform *Wudu*—a ritual washing in preparation for prayer.	**Awareness—**Muslims may want to perform a daily prayer. **Practical actions: Signposting**—Become aware of the availability and location of a prayer room and an appropriate washing area within the Trust or organisation. *If not available;* **Temporal prayer space** – if possible provide a temporal quiet space for the prayer to be performed. May provide a clean sheet for them to perform the prayer on.
**Modesty and privacy**		
In Islamic ethics modesty is reflected in a Muslim’s speech, dress and conduct. Outward modesty is often reflected in how a Muslim woman dresses in the presence of other people or the opposite gender. This can vary between Muslim women, from wearing clothing that is not revealing to full covering including the head and sometimes the face.	**Covering**—may need support in limiting how much of her body is uncovered, during clinical examination, regular maternal scans, while wearing a theatre gown, or while being in hospital wards and during labour or while breastfeeding. **Preference for a female HCP—**some Muslim women prefer not to be attended to or examined by a male HCP, or only if absolutely necessary. This includes male student trainees or male interpreters. Even in an emergency, some Muslim women may still refuse to be attended to by a male HCP.	**Awareness—**Muslim women may want to maintain modesty and limit body exposure e.g. during clinical examination. This can include covering certain body parts (including head and face) and/or being attended to by a female HCP. **Practical actions:** **Provide resources** – If possible, provide additional sheets, or stockings to cover legs or provide an extra gown, for example, during a maternal scan or in labour to support Muslim women to feel comfortable and not too exposed. **Flexibility**: During the preparation for theatre, walking or getting to theatre, wearing a short hospital gown can be uncomfortable for some Muslim women. If possible, allow women to keep their headscarf or their overall long garment on until they get to theatre, removing this once in theatre room. Alternatively, provide an extra sheet/gown or stockings to cover legs. Ask a woman if she prefers her curtains to be open or closed e.g. during visiting hours or while she is breastfeeding **Provide time for the women to cover-** While in a private appointment, examination or labour room some Muslim women may have taken their headscarf or face veil off or removed certain clothing—exposing their body. Give time for women to replace garments, for example, a headscarf before moving them to a different room, opening bay curtains or allowing another HCP into the room. **Being informative** – make the woman aware that she is going to be consulted or attended to by a male HCP. If possible, explore if the woman is comfortable and if she has other preferences. If a female HCP is preferred, check if this can be organised. If not, ensure that this is explained to the woman, especially, in an emergency situation- highlighting that the key objective at this time is her safety. This will help the woman make an informed decision.
**Dietary considerations**		
It is common for Muslims to consume and accept what is considered lawful (HALAL) by Islamic teachings and to refrain from anything considered prohibited (HARAM). Main prohibited foodstuffs and liquids include Pork (and its by-products); animal fats and meat that has not been slaughtered according to Islamic teachings and alcohol. There are certain exceptions to these teachings that allow for animal-based medications or vaccine to be used e.g. if there is no other lawful option available and their usage is for the greater benefit of the person. *Note: not all Muslim women may consider this religious exception*	**Alternative options—**Some Muslim women may reject certain medication or vaccines (e.g. Vitamin-K for the new-born) if they contain any unlawful substance. **Being informed**: Muslim women expect to be informed of the content of medication prescribed.	**Awareness—**some Muslim women may require alternative options that adhere to their religious teaching. **Practical actions**: **Record specific dietary requirement**- explore with the woman any specific dietary requirement and their preferences if alternatives options are not available. **Flexibility with alternative options-** e.g. when providing food, note that not all Muslim women will be happy with a HALAL option; some may prefer something else such as a vegetarian option. **Being aware-** it is important to be knowledgeable about content of medication or vaccine provided (whether it contains any animal extracts within it). It is important that women are informed and are given a choice if another suitable option is available. *Note: this is also important for other women who may be vegetarians, vegans, Hindu, Jews and others with specific dietary requirements.* **Provide possible alternative**- if available provide possible alternatives e.g. medications that are suitable for vegetarians or vegans are often an appropriate alternative.
**Fasting in the month of Ramadhan**		
It is common for Muslims to fast during the Islamic month of Ramadhan- often described as a community worship, it includes observing fast (no eating or drinking) from sunrise to sunset. *Note: There is a religious exception for a pregnant or breastfeeding woman who can choose not to observe fast during the month of Ramadhan but make up the fast at a different time of the year.*	**Open discussion—**Some Muslim women who are pregnant or breastfeeding during the month of Ramadhan may attempt to fast. They may not want to miss out on the community fast and find motivation to engage in the fast with the rest of their family. Muslim woman may need to have a discussion with the HCP to help them make an informed decision. **Nutritional advice—**may require nutritional advice to support them during the fast.	**Awareness—**some Muslim women may observe fast during the month of Ramadan. **Practical actions:** **Ramadhan on the annual calendar –** It is important to be aware of when the month of Ramadhan is. *Note: some Muslim women may not feel confident to mention their intention to fast.* **Open discussion**—Telling Muslim women not to fast can often inhibit discussion. It is important to explore fasting with the woman; allowing for discussion of her intentions during the month of Ramadhan, and how she is finding the fast if she is fasting. **Nutritional advice** – Provide guidance to help a woman keep healthy during this period. Refer woman to nutritionist if necessary to help guide her during this period.
**Quran Recitation**		
The recitation of the Quran is the first and most constant religious practice carried out by Muslim women throughout the stages of childbirth and during pregnancy—reflecting on the words of Allah and exposing their unborn child to hearing the word of Allah.	**Aware of opportunity** – a woman may not be aware if it is possible for her to play her Quran recitation during her stay in hospital, labour or in theatre. **Resource available**—some women will use their own devices to play the Quran recitation (e.g. mobile phone) others may require a device.	**Awareness—**Be aware that some women may recite Quran during their stay in hospital including whilst in theatre or during labour. **Practical action:** **Open discussion-** explore with the woman any specific requirement during labour or in theatre e.g. explain to the woman that she has the option to play the recitation of the Quran during labour or in theatre (if possible). **Provide resource** – If possible, provide a CD player or audio player to play their Quran recitation or provide headphones to help them use their own devices.
**Birth position**		
Some Muslim women do not wish to be in a laying down position during labour– preferring instead to imitate Maryam (Mary the mother of Jesus) during her birth.	**Support during labour** – Requires HCPs awareness of women’s preference in order to support women during labour to get into the position they prefer.	**Awareness—**Discuss preference while exploring the birth plan with the woman, e.g. explore if there is anything specific they would like to practice and keep this on record. **Practical action**: **Provide recourses –** to support the woman during labour to get into the position she prefers (if safe).
**Silent birth**		
Some Muslim women prefer the first word that their child hears at birth to be Allah’s name or the word of Allah.	**Acknowledgment**—some women prefer a moment of silent at the point of birth, so that they can mention Allah’s name for the child to hear. If not silent, some women may call the word of Allah at birth slightly louder than the other voices in the room for it to be significant for the child to hear than the other voices.	**Awareness and acknowledgment—**some women prefer a moment of silent at the point of birth or request HCP to speak quietly during the birth. **Practical actions**: **Open discussion** – while exploring the birth plan with the woman and her preferences during labour, check if she would like silent birth and record in notes. **Silent birth**—if possible, during the birth try to minimise speaking as much as possible to give the chance for the mother and birth partner to speak the first words they want the child to hear. Women having caesarean section may request this too.
***Adhan* and Iqamah (call for prayer)**		
To whisper the *Adhan* to the new-born soon after birth. These words include the name of Allah the Creator and is followed by the Declaration of Faith: “*There is no deity but Allah; Muhammad is the Messenger of Allah*.” It is customary for the father, or a respected member of the family or local community, to whisper the Adhan. The ceremony takes only a few minutes.	**Acknowledgment** – often the birth-partner (father or a mother figure) will do this practice, they may move to one side of the room with the baby to have a personal moment and some may wait until there is no interruption in the room. Some may wait for a member of the family or community with high status (such as grandfather) to carry out this practice.	**Awareness—**parents my request to have the baby and some privacy after birth to perform this practice. **Practical actions**: **Provide some one-to-one time with the baby—**if possible, give parents some one-to-one time with the baby following the birth. Silence may be required in the room to perform the *Adhan.* **Flexibility in access—**restricting access to the delivery ward to partners can affect this practice.
**Burial of placenta**		
Out of respect, women are encouraged to bury any separate part of the human body if possible. *Note: not all Muslim women would engage in this practice.*	**Being informed:** Some Muslim women may not be aware of the options available with regard to taking their placenta home. **Guide and instructions**: Some Muslim women may have the facility to bury their placenta and others may not. Women need information about how to safely transport and dispose of their placenta if they decide to take it home.	**Awareness—**some Muslim women may request to keep their placenta. *Note: some Muslim women may not feel confident to ask if they are not aware of this option.* **Practical action:** **Provide the option—**make sure that all women are aware of this option. If this option is not available due to, for example, further tests being required on the placenta, women need to be informed. **Provide guidance** – Provide guidance e.g. in a leaflet with appropriate and understandable language that can be read through with the woman to ensure that she understands the instructions.
**Breastfeeding**		
It is religiously recommended for a woman to breastfeed for a period of 2 years. *Note: The majority of Muslim women will have the intention to breastfeed and may attempt breastfeeding but my struggle to continue if not supported.*	**Support initiation and continuing breastfeeding** – Women need support in initiating and continue breastfeeding. This includes hands on and follow up support from breastfeeding support team. **Maintaining modesty** – Some Muslim women may not feel confident to breastfeed in hospital wards, if for example, bay curtains are open or breastfeeding in front of other women. **Reassurance** – some Muslim women are keen to fulfil their religious recommendation of breastfeeding, however, may struggle and lose confidence in their ability to breastfeed.	**Awareness—**that some Muslim women will be keen to breastfeed but may not be aware of some of the challenges they may face during this period. **Practical actions**: **Open discussion** – give time during pregnancy to explore the woman’s intention in breastfeeding and discuss what is important and any support she may require. **Support to initiating breastfeeding** – offer hands on support to help women initiate breastfeeding. If in a situation where the new-born is away from the mother due to clinical reason (e.g. child needs to be in the incubator), explore possible options to support mother to breastfeed e.g. support women to express her breast milk. **Consent to donated breast milk**—Muslim women may object to using donated breast milk due to strict religious implications e.g. if breast milk is ever shared with another child, then the two children are considered to be siblings and would not be allowed to marry each other when older. Women need to be consulted and well informed if donated breast milk is to be a suggested offer. **Support privacy during breastfeeding**—some women may prefer their curtains to be closed while in a ward to help them breastfeed and a breastfeeding apron can support women maintain their modesty. Allow time for women to appropriately cover themselves while breastfeeding before allowing other HCPs or visitors into the room. **Continues checks**—connect women to breast feeding support team to ensure that she is getting the support needed. Check with the woman during home visits and 6-week check if she requires further support **Provide reassurance** – some women may find that breastfeeding is affecting them emotionally and mentally. It’s important that this is discussed with the woman and the best ways to support her are explored.
**Male Circumcision**		
This is a religious obligation practiced by the majority, if not all Muslims, as it is for the Jewish community. It is recommended that circumcision is performed within the first few weeks of life. Since this is not available on the NHS, some Muslims will seek out private clinics and some will travel to their home country to have this done. Other women prefer to delay circumcision until the child is of an age (over 1 years old) when the procedure may be available within some NHS Trusts.	**Information about options available on the NHS –** women may need guidance about NHS recommendations with regard to male circumcision. **Alternative options**—difficulties in obtaining circumcision on the NHS means that it is usually performed in the private sector. Women may need advice on where is safe e.g. information about NHS accredited private clinics.	**Awareness—**that this is a practice that Muslim women are likely to implement. *Note: women may not feel confident to discuss this with HCPs due to the controversy around this practice.* **Practical action:** **Open discussion –** Let the parent know that you are aware of this religious practice and have an open discuss about parents’ intentions and what support they need. This will include discussion about safety of the child, especially in infants that have clinical issues. For example, discuss delay of circumcision in jaundiced infants due to potential risk of prolonged bleeding, or in infants born with hypospadias where surgical opinion has been sought. **Provide information**—be aware of the NHS recommendations around male circumcision to help guide parents to make informed decisions. **Signposting-** if possible, get a list of NHS accredit private clinics that you can share with parents to support them pursue a safe option for their child.

This study provides an in-depth insight into Muslim women’s religious practices, promoting better communications and interactions between women and health care professionals. Study findings also support Rassool’s suggestion to support healthcare professionals to develop levels of awareness, skills and religion/cultural sensitivity that can be applied to interactions with Muslim patients and their family [3].

### Strengths and limitations

This study is the first of its kind to develop evidence-based recommendations concerning the care of Muslim women during pregnancy, based on the lived experiences and perceptions of Muslim women and HCPs. A Key strength of this study is that it presents details of universal Islamic religious practices narrated within Islamic teachings and tradition that have been practiced overtime and reflecting on how Muslim women in the UK experiences these practices. Also, this study has included the involvement of members of the public (including a Muslim woman and Muslim HCPs) who informed the recommendations.

Use of these recommendations in practice and their impact on quality of care for Muslim women has yet to be explored. Religious practices in relation to stillbirth and neonatal deaths were not explored in this study, further exploration in this respect is needed to add to current recommendations.

## Conclusion

The study recommendations promote a woman-centred approach that takes in to account Muslim women’s specific needs as defined by them. It is hoped that these recommendations will facilitate conversations between Muslim women and HCPs that will help address the individual needs of Muslim women.

## Data Availability

The datasets generated and/or analysed during the current study are not publicly available as they may contain information that could compromise the confidentiality and anonymity of the participants but are available (limited) from the corresponding author on reasonable request.

## Abbreviations

HCPs: Healthcare professionals
